# Association of IL-9 Cytokines with Hepatic Injury in *Echinococcus granulosus* Infection

**DOI:** 10.3390/biom14081007

**Published:** 2024-08-14

**Authors:** Tanfang Zhou, Xinlu Xu, Jiang Zhu, Mayire Aizezi, Aili Aierken, Menggen Meng, Rongdong He, Kalibixiati Aimulajiang, Hao Wen

**Affiliations:** 1 State Key Laboratory of Pathogenesis, Prevention and Treatment of High Incidence Diseases in Central Asia, Clinical Medicine Institute, The First Affiliated Hospital of Xinjiang Medical University, Urumqi 830011, China; fangtan1105@163.com (T.Z.); xuxinlu62@163.com (X.X.); doctor_zhujiang@163.com (J.Z.); 13199829372@163.com (M.A.); ali198908@163.com (A.A.); menggen2023@163.com (M.M.); herongdong308@126.com (R.H.); 2 Digestive and Vascular Surgery Center Therapy Center, Department of Hepatobiliary and Hydatid Disease, The First Affiliated Hospital of Xinjiang Medical University, Urumqi 830011, China; 3 Department of Abdominal Surgery, The Third People Hospital of Xinjiang, Urumqi 830000, China

**Keywords:** IL-9, TLR2, cystic echinococcosis, *Echinococcus granulosus*, liver injury, immunity

## Abstract

Cystic echinococcosis (CE) is a zoonotic disease caused by the parasite *Echinococcus granulosus* (*E. granulosus*), which can lead to the formation of liver lesions. Research indicates that *E. granulosus* releases both Toll-like receptor 2 (TLR2) and Interleukin-9 (IL-9), which can potentially impair the body’s innate immune defenses and compromise the liver’s ability to fight against diseases. To investigate the role of TLR2 and IL-9 in liver damage caused by *E. granulosus* infection, samples were initially collected from individuals diagnosed with CE. Subsequently, BALB/c mice were infected with *E. granulosus* at multiple time points (4 weeks, 12 weeks, 32 weeks) and the expression levels of these markers was then assessed at each of these phases. Furthermore, a BALB/c mouse model was generated and administered anti-IL-9 antibody via intraperitoneal injection. The subsequent analysis focused on the TLR2/MyD88/NF-κB signaling pathway and the expression of IL-9 in *E. granulosus* was examined. A co-culture experiment was conducted using mouse mononuclear macrophage cells (RAW264.7) and hepatic stellate cells (HSCs) in the presence of *E. granulosus* Protein (EgP). The findings indicated elevated levels of IL-9 and TLR2 in patients with CE, with the activation of the signaling pathway significantly increased as the duration of infection progressed. Administration of anti-IL-9 in mice reduced the activation of the TLR2/MyD88/NF-κB signaling pathway, exacerbating liver injury. Moreover, EgP stimulates the TLR2/MyD88/NF-κB signaling pathway, resulting in the synthesis of α-SMA and Collagen I. The data suggest that infection with *E. granulosus* may stimulate the production of IL-9 through the activation of the TLR2/MyD88/NF-κB signaling pathway, which is mediated by TLR2. This activation stimulates RAW264.7 and HSCs, exacerbating liver injury and fibrosis.

## 1. Introduction

Cystic echinococcosis (CE) is a parasitic disease primarily affecting the liver and is highly prevalent in *Echinococcus granulosus* (*E. granulosus*) in Northwest China. Despite the high global prevalence of CE and generally modest fatality rates, the lack of effective prevention and control measures can significantly increase mortality. Thus, CE remains a significant public health concern [1]. CE is a zoonotic disease that affects the liver. The mature parasite resides in the small intestine of its definitive host, typically canines, and produces infectious eggs. When humans, as accidental intermediate hosts, ingest these eggs, the larvae emerge and cause inflammation, varying degrees of damage, and fibrosis in the liver [1,2]. Investigating the relationship between parasites and their hosts is an active area of research. Parasites can partially modulate the host’s immunological response and evade immune detection, allowing them to survive successfully in the host.

The immune system’s defense against CE is primarily categorized into adaptive and innate responses [3]. The innate immune system acts as the primary line of defense against infectious pathogens and parasites. Pattern recognition receptors (PRRs) are a fundamental component of this system, as they are responsible for identifying a multitude of pathogen-associated molecular patterns. Toll-like receptors (TLRs), a type of PRR, play a pivotal role in activating immune cells, such as macrophages and dendritic cells, by recognizing specific molecular patterns derived from bacteria and parasites [4]. MyD88 is the primary signaling molecule closely linked to the TLRs family. It facilitates the activation of mitogen-activated protein kinase (MAPK) through a signaling pathway dependent on MyD88 following TLRs activation. This leads to the induction of NF-κB, which translocate to the nucleus and initiates the immune response [5]. Existing research suggests a potential association between TLRs and Echinococcus infection. These receptors can recognize and respond to antigens derived from helminths, providing new insights into the immune response of the host to parasitic infection [6,7,8].

TLR2 is a notable PRR that can form homodimers or heterodimers with TLR1 or TLR6. It can recognize specific pathogen-associated molecular patterns (PAMPs) linked to infections. TLR2 plays a crucial role in detecting a wide range of microorganisms and initiating the host’s immunological response, particularly in combating parasitic diseases. Previous studies have shown that TLR2 plays a significant role in the onset and progression of liver disorders, and in regulating cellular function through distinct signaling pathways. For example, HBsAg can stimulate RAW264.7 through the TLR2/MyD88/NF-κB signaling pathway, enhancing the mobility, proliferation, and contraction of HSCs, thereby exacerbating liver fibrosis [9]. Additionally, preliminary research has indicated increased TLR2 activation in individuals infected with *E. granulosus,* suggesting that TLR2 may play a critical role in regulating the invasion and long-term survival of parasites within human hosts [7]. Concomitantly, CE infection may upregulate anti-inflammatory cytokines via TLR2-mediated pathways, thereby suppressing the host’s immune responses. The immune regulatory responses during CE infection may be associated with TLR2 signaling [10,11,12]. Therefore, it can be proposed that TLR2 may have a detrimental effect on the progression of *E. granulosus* infection and may also enhance the immune response associated with this infection via the TLR2/MyD88/NF-κB signaling pathway.

IL-9 is a multifaceted cytokine synthesized by a diverse array of immune cells, including mast cells, NKT cells, Th2 cells, Th17 cells, regulatory T (Treg) cells, and Th9 cells. Th9 cells are the primary producers of IL-9 [13,14]. To date, IL-9 has been shown to play a role in the development of several diseases, including cancer, autoimmune diseases, and immunomodulatory diseases caused by pathogens. IL-9 profoundly influences the immune response to parasites and is implicated in the pathogenesis of allergic disorders, including asthma and bronchial hyperresponsiveness [14,15]. IL-9 was initially identified in cases of gastrointestinal nematode infection and allergic asthma [16,17,18,19,20,21]. Previous research has demonstrated a significant reduction in IL-9 levels following surgical intervention and albendazole treatment for CE, suggesting that IL-9 may be involved in the immune response triggered by *E. granulosus* infection [22]. Furthermore, evidence suggests that IL-9 may exacerbate the inflammatory response in the liver of patients with CE by activating the TGF-β/Smad signaling pathway and enhancing IL-9 production in individuals with active CE [23]. Therefore, it can be hypothesized that IL-9 may play a role in the development of *E. granulosus* infection.

It has been demonstrated that the activation of TLR2 on CD4^+^ T cells stimulate the development and activity of Th9 cells [24]. However, the precise mechanism by which IL-9 and TLR2 interact in *E. granulosus*-infected mice and their impact on liver injury is not yet fully understood. Further research is needed to determine whether inhibiting IL-9 and modulating TLR2 signaling exacerbates liver damage in mice infected with *E. granulosus*. The objective of this study was to investigate the immune response mechanism in *E. granulosus*, with a specific focus on assessing the impact of IL-9-regulated TLR2 signaling as a potential molecular target for improving disease immunological control and vaccine efficacy.

## 2. Material and Methods

### 2.1. Patients

The study was conducted at the First Affiliated Hospital of Xinjiang Medical University from May to October 2023. A total of 40 participants were enrolled, including 20 healthy controls (HC) and 20 patients with CE, classified according to WHO Informal Working Group on Echinococcosis (WHOIWGE) criteria [25,26]. Clinical characteristics of enrolled subjects are shown in Table 1. One specimen was taken close to the parasitic lesion, including the metacestode (CLT, ‘close liver tissue’, ~0.5 cm from lesion), and another was taken from the macroscopically normal liver distant from the lesion (DLT, ‘distant liver tissue’, at least 2 cm from the lesion), as described in our research team’s previous publication [27]. Peripheral blood samples (PB) were obtained from 20 CE patients and 20 healthy age- and sex-matched controls. The study was approved by the hospital’s Ethics Committee (Approval NO. 20211015-53).

### 2.2. Mice

Female BALB/c mice, aged 8–10 weeks and weighing 20 ± 2 g, were obtained from the Medical Laboratory Animal Center of Xinjiang Medical University, China. The mice were housed in a specified pathogen-free (SPF) environment with controlled temperature, humidity, and a 12-h light/dark cycle. They had unrestricted access to food and water. The study adhered to the Laboratory Animal Center of Xinjiang Medical University’s criteria and was approved by the Institutional Animal Care and Use Committee (Approval NO. 20230321-14).

### 2.3. Cells

RAW264.7 cells were thawed and cultured in Pricella medium (Wuhan, China) at 37 °C with 5% CO_2_. Cryopreserved hepatic stellate cells (HSCs) were revived using a mixture of 45 mL basal medium, 5 mL 5% fetal bovine serum (Vivacell, Shanghai, China), and 500 μL 1% double antibody (Vivacell, Shanghai, China) in 1640 medium (Vivacell, Shanghai, China). Both cell types were plated in six-well plates at a density of 1 × 10^6^ cells per well. After 24 h, a solution containing 2 μL of protein (15 μg/mL) was added to the medium, followed by 2 μL of the TLR2 agonist (Diprovocim, HY-123942, 500 nM, MCE, Monmouth Junction, NJ, USA) and 2 μL of the TLR2 inhibitor (C29, HY-100461, 50 μM, MCE, Monmouth Junction, NJ, USA). Negative controls (NC) were injected with an equivalent volume of PBS. Each condition was tested in three wells, with the experiment repeated three times. At the end of the experiment, cell samples were collected, and RNA and protein were extracted for further analysis.

### 2.4. Preparation of E. granulosus and EgP

*E. granulosus*-infected sheep liver was obtained from a slaughterhouse in Urumqi, China [28]. PSCs were treated with 1% pepsin in saline at 37 °C for 30 min, with the pH adjusted to 3.0 [29]. PSCs were separated from the liquid portion through repeated suspension, filtration, and precipitation with normal saline. The collected PSCs were pulverized using a grinder (Servicebio, Wuhan, China) and mixed with two-thirds of the volume of PBS, then incubated overnight on a shaker at 4 °C. The next day, the mixture was centrifuged at 12,000 rpm for 30 min at 4 °C. The supernatant was collected, and protein concentration was determined using the bicinchoninic acid (BCA) protein assay (Solarbio, Beijing, China). The protein samples were aliquoted into 1.5 mL EP tubes and stored at −80 °C for future use.

### 2.5. Mouse Model of E. granulosus Infection and In Vivo Blockade

Mice were inoculated via the hepatic portal vein with live PSCs in saline as previously described [27,30,31], whereas control mice were injected with isotonic saline. The mice were randomly divided into six groups of six: 4-week, 12-week, and 32-week infection groups, each with corresponding control groups. To induce infection, 2000 PSCs of *E. granulosus* were injected into the hepatic portal vein (p.v.) under anesthesia via laparotomy. Sham surgery group mice received PBS injections. Mice were euthanized at 4, 12, and 32 weeks for analysis. Additionally, three experimental groups of six mice each were established: a control group, a model group, and an intervention group. Mice in the experimental group were infected with *E. granulosus* and received intraperitoneal injections (i.p.) of isotype control antibody (100 μg, mouse igG2a, clone C1.18.4, BioXcell). Mice in the intervention group received anti-IL-9 mAb (100 μg, clone 9C1, BioXcell). Treatments were administered via intraperitoneal (i.p.) injection every three days for twelve weeks, starting three days after infection. At the end of the study, liver samples from 18 mice were collected for analysis.

### 2.6. Preparation of PBMCs

In the early morning following admission, blood samples were collected from both healthy individuals and patients into vessels containing 0.2 mL of heparin sodium to prevent clotting. The blood samples were mixed with an equal volume of PBS and agitated to ensure uniform cell distribution. An equal volume of lymphocyte solution (Solarbio, Beijing, China) was added to the diluted blood, and the mixture was subjected to density gradient centrifugation. After centrifugation, the opaque white layer of lymphocytes in the middle was collected. The cells were then lysed to obtain pure human peripheral blood mononuclear cells (PBMCs). These isolated PBMCs were subsequently used in qRT-PCR studies to evaluate specific gene expression levels.

### 2.7. Quantitative Real-Time Polymerase Chain Reaction (qRT-PCR)

Extract total RNA from mouse liver tissue and collected cells, lyse with Trizol reagent, and then quantify it. The mRNA was then converted into complementary DNA (cDNA) using the HiScript III RT SuperMix for qPCR (Vazyme, Nanjing, China). The cDNA was analyzed via qRT-PCR using the ChamQ SYBR qPCR Master Mix (Vazyme, Nanjing, China) on a qPCR machine (Bio-Rad, Foster City, CA, USA). Specific forward and reverse primers, synthesized by Sangon (Shanghai, China) and listed in Table 2, were used for the qRT-PCR. The GAPDH gene served as an internal reference to ensure accuracy and comparability of the results.

### 2.8. Western Blot

Proteins were isolated from mouse liver tissue, RAW264.7 cells, and HSCs. The protein concentration was quantified using the BCA protein quantification kit (Solarbio, Beijing, China) to ensure accuracy. SDS-PAGE (Biotides, Beijing, China) was used for protein separation, followed by transfer to PVDF membranes (Millipore, Billerica, MA, USA). The membranes were blocked with 5% BSA for 2 h and then incubated overnight at 4 °C with specific primary antibodies. After washing with TBST, the membranes were incubated with HRP-conjugated secondary antibodies (multi-rAb HRP-goat anti-rabbit, 1:5000, RGAR001, Proteintech, Wuhan, China) and detected using an enhanced chemiluminescence (ECL) kit (Biosharp, Hefei, China). Primary antibodies included anti-rabbit IL-9, 1:1000, ab227037, Abcam; anti-rabbit NF-κB p65, 1:1000, ab16502, Abcam, Cambridge, UK; anti-rabbit MyD88, 1:200, 67969-1-Ig, Proteintech; anti-rabbit anti-α-SMA, 1:8000, 80008-1-RR, Proteintech; anti-rabbit Collagen I, 1:2000, 14695-1-AP, Proteintech; and anti-rabbit GAPDH, 1:5000, GB15004-100, Servicebio, Wuhan, China, which was used as a control.

### 2.9. Enzyme-Linked Immunosorbent Assay (ELISAs) and Liver Enzyme Assays

The serum of the subjects was collected, and the levels of TLR2, IL-9, MyD88, and NF-κB p65, as well as alanine aminotransferase (ALT) and aspartate aminotransferase (AST) were measured. Blood samples from mice at various time points following *E. granulosus* infection were collected, and the levels of TLR2, IL-9, MyD88, and NF-κB p65 were measured. The concentrations of cytokines were measured using ELISA kits according to the manufacturer’s instructions (Lapuda, Nanjing, China).

### 2.10. Hematoxylin and Eosin (H&E) and Periodic Acid-Schiff (PAS)

Mouse liver specimens were fixed in 4% tissue fixation solution (Biosharp, Hefei, China) to preserve tissue structure. The specimens were then processed, embedded, and sectioned into paraffin slices for staining and microscopic examination. Hematoxylin and eosin (H&E) staining and periodic acid-Schiff (PAS) staining was performed using the staining kit (Solarbio, Beijing, China). H&E staining allows for the assessment of inflammatory cell infiltration, such as lymphocytes and neutrophils, under high magnification, as well as the evaluation of hepatocellular damage, including cellular necrosis, pyknotic nuclei, karyorrhexis, and cellular vacuolation. PAS staining assesses liver pathology by evaluating the extent of hepatocyte necrosis, the degree of inflammatory cell infiltration, and the abnormal deposition of glycogen within hepatocytes. The stained slices were examined under a light microscope (Olympus, Tokyo, Japan), and images were captured for further analysis and documentation.

### 2.11. Immunohistochemical Staining (IHC)

Liver tissues (5 μm) from both human and mouse samples were fixed in 4% tissue fixation solution (Biosharp, Hefei, China), embedded in paraffin, and sectioned. The sections were dewaxed with water, and xylene was removed using a gradient alcohol solution. The sections were then subjected to heat-mediated antigen retrieval in Tris-EDTA buffer (Solarbio, Beijing, China) and sodium citrate buffer. Subsequently, the endogenous peroxidase activity was inhibited using 3% H_2_O_2_. The sections were blocked for 1 h at room temperature in PBS with 10% goat serum (blocking buffer) and then incubated overnight at 4 °C with primary antibodies in blocking buffer (anti-rabbit TLR2, 1:200, 17236-1-AP, Proteintech; anti-mouse MyD88, 1:250, 67969-1-Ig, Proteintech; anti-mouse IL-9, 1:100, 666144-1-Ig, Proteintech, Wuhan, China; Anti-rabbit NF-κB p65, 1:2000, ab16502, Abcam, Cambridge, UK). The next day, unbound primary antibodies were removed by washing with PBS. Sections were then incubated with secondary antibodies (goat anti-rabbit, Proteintech, Wuhan, China) at 37 °C for 1 h. Color development was achieved using 3, 3′-diaminobenzidine (DAB) (Proteintech, Wuhan, China) at a dilution of 1:20. The stained sections were then examined and photographed at 200× magnification (3 fields/section/sample) using a digital image acquisition system (Olympus, Tokyo, Japan). The negative control is prepared without the addition of the primary antibody and replaced with goat serum to detect the non-specific binding of the secondary antibody. The positive control uses tissue sections known to express the target protein to demonstrate the presence of the antigen of interest. IL-9 is predominantly expressed in the cell nucleus, with the rest expressed in the cytoplasm. The statistical outcome is determined by the percentage of positive cells relative to the total number of cells in the field of view.

### 2.12. Fluorescence-Based Multiplex Immunohistochemistry (mIHC) Staining

Tissue sections were first dewaxed by removing paraffin with xylene and rehydrated with a gradient of ethanol. High-temperature antigen retrieval was performed using a Tris-EDTA buffer and sodium citrate buffer to expose antigen sites. Endogenous peroxidase activity was blocked with a 3% H_2_O_2_ solution. Sections were then incubated overnight at 4 °C with primary antibodies (anti-rabbit TLR2, 1:200, 17236-1-AP, Proteintech; anti-mouse IL-9, 1:100, 66144-1-Ig, Proteintech, Wuhan, China). The following day, sections were washed with PBS and incubated with secondary antibodies for 50 min at room temperature. Tyramide signal amplification (TSA) reagent (Servicebio, Wuhan, China) was used to enhance signal detection. For triple staining, antigen retrieval and blocking steps were carried out as described. This was repeated for up to three antibodies. Finally, nuclei were stained with DAPI (Servicebio, Wuhan, China) for visibility. Slides were sealed with neutral gum to prevent drying or contamination and examined using a fluorescence microscope (Nikon, Tokyo, Japan) for computerized quantification.

### 2.13. Data Analysis

The laboratory offers the results of proteomic and transcriptomic studies. To create a Protein-Protein Interacting (PPI) network, we utilized the Search Tool for Retrieval of Interacting Genes/Proteins (STRING) database, accessible at https://STRING-db.org (accessed on 28 April 2024). Additionally, to identify biological pathways, we consulted the Kyoto Encyclopedia of Genes and Genomes (KEGG) database. Supplement Figures S1 and S2 display the outcomes of the analysis obtained from the publicly accessible database, accessible at https://david.ncifcrf.gov/ (accessed on 28 April 2024).

### 2.14. Statistical Analysis

The statistical analysis of this study was conducted using the statistical software SPSS 26.0. The data were subsequently subjected to statistical analysis using Prism version 9.0. The data are presented as the mean value, with the standard deviation indicated by the plus or minus symbol. To evaluate significant differences among various experimental groups, we employed either t-tests or one-way analysis of variance (ANOVA) using data obtained from at least three independent trials. The correlation analysis was conducted using Spearman’s correlation coefficient. In this investigation, statistical significance was defined as *p*-value < 0.05.

## 3. Results

### 3.1. The Expression of TLR2 and IL-9 Increased in CE

**Figure 1:**
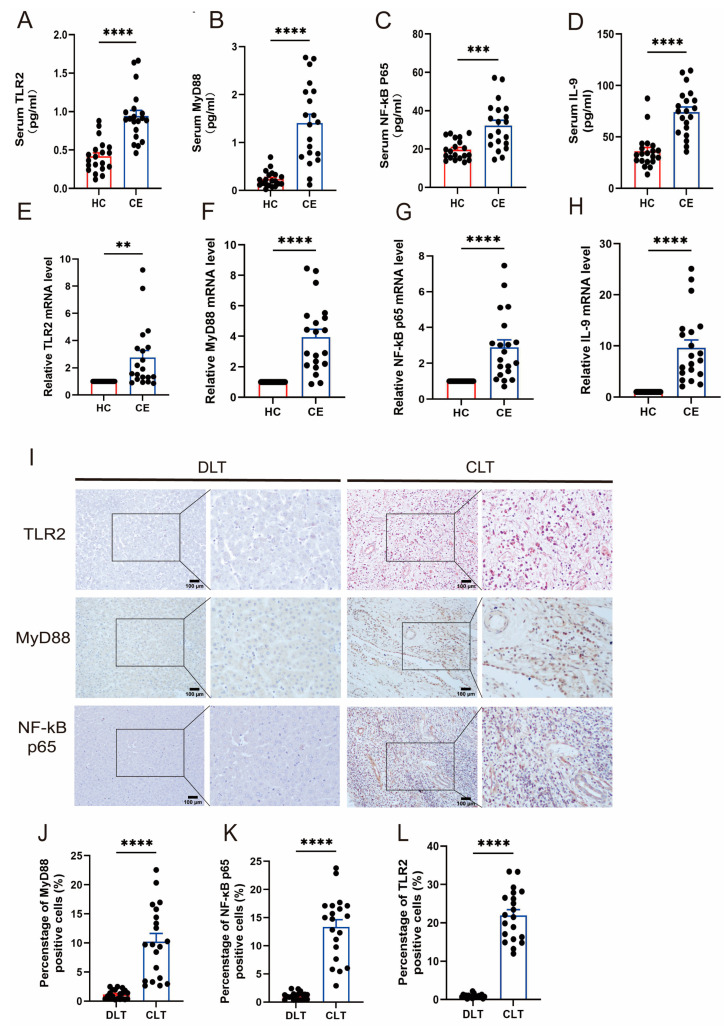
The expression of TLR2 and IL-9 increased in CE. To confirm this, we used the ELISA method to accurately measure the levels of TLR2 (**A**), MyD88 (**B**), NF-κB p65 (**C**), and IL-9 (**D**) in serum samples obtained from a group of HC (n = 20) and another group of patients with CE (n = 20). Furthermore, PBMCs were extracted from the plasma of both groups, and the expression levels of TLR2 (**E**), MyD88 (**F**), NF-κB p65 (**G**), and IL-9 (**H**) mRNA were examined by RT-qPCR. In addition, we performed IHC labeling of TLR2, MyD88, and NF-κB p65 on liver sections obtained from both HC and CE groups. The resulting images (**I**) were magnified 200×, with the left side showing the overall view and the right side showing a close-up view. The percentage of positively stained cells was calculated to assess the expression of TLR2 (**J**), MyD88 (**K**), and NF-κB p65 (**L**) (n = 20). Statistics are displayed as the mean ± SD. ** *p* < 0.01, *** *p* < 0.001 and **** *p* < 0.0001.

The initial study of the database and Protein Interaction Network (PPI-RRB) indicated a significant correlation among TLR2, MyD88, and IL-9. Detect serum levels of AST and ALT and perform a correlation analysis with TLR2 and IL-9 (Figure S1A–E), with the imaging pictures shown in Figure S1F. The findings suggest that these proteins play a pivotal role in biological signaling, gene expression regulation, energy metabolism, and cell cycle regulation (Figure S2A). The three-dimensional structural diagram of the protein provides a visual representation that aids in understanding its function and biological mechanism (Figure S2B). A study was conducted using the KEGG database to investigate the primary metabolic regulatory pathways of the TLR2/MyD88/NF-κB signaling system. This research provided a comprehensive understanding of the significance of these pathways in disease (Figure S2C). The study involved 40 participants, including 20 patients with CE and 20 HC. The concentrations of TLR2 (Figure 1A), MyD88 (Figure 1B), NF-κB p65 (Figure 1C), and IL-9 (Figure 1D) were quantified in the serum of the participants. The serum levels of these markers were significantly elevated in individuals with CE compared to healthy controls. Furthermore, the comparative levels of TLR2 (Figure 1E), MyD88 (Figure 1F), NF-κB p65 (Figure 1G), and IL-9 (Figure 1H) were markedly elevated in patients with CE compared to healthy individuals. The immunohistochemical data corroborated our findings. The expression of TLR2 and the TLR2 signal pathway in the CLT was considerably greater than in the DLT (*p* < 0.0001) (Figure 1J–L). Consequently, the levels of TLR2 and IL-9 in CE exhibited a significant increase, suggesting a potential correlation between IL-9 and TLR2 in CE. It is postulated that CE may exert an immunomodulatory effect on the liver by enhancing the activity of the TLR2 signaling pathway.

### 3.2. The Expression of TLR2 Increased with the Time of Infection

**Figure 2:**
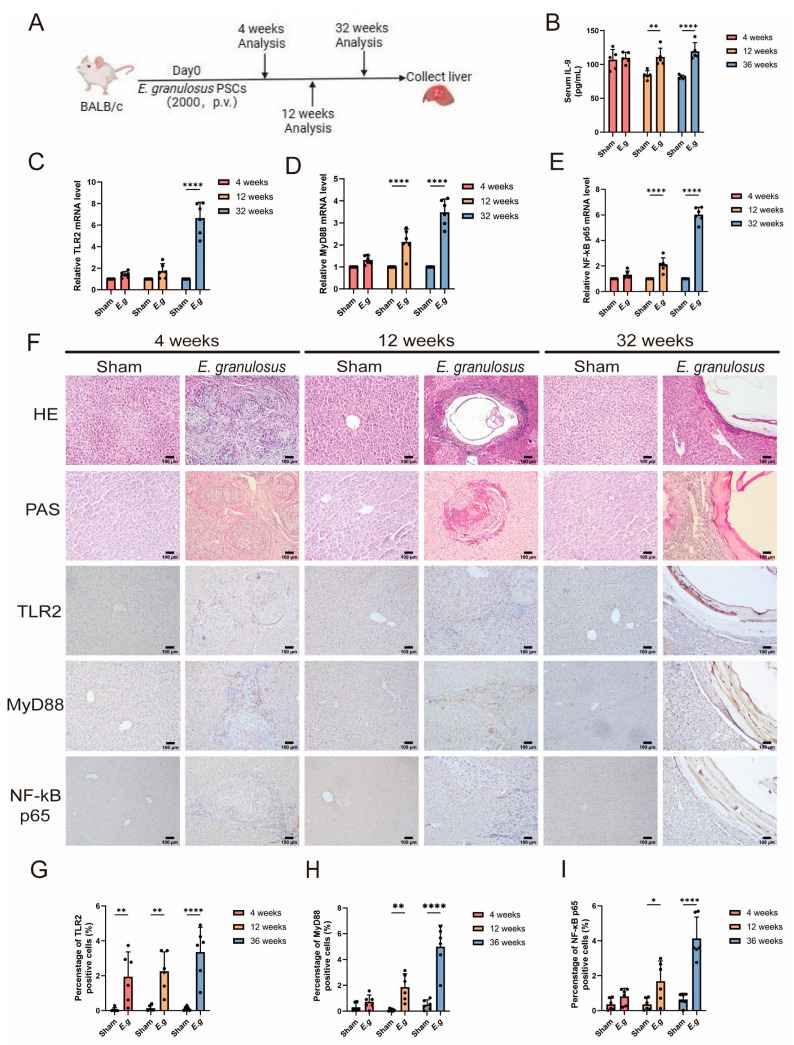
The expression of TLR2 increased with the time of infection. For this investigation, we selected female BALB/c mice and established a mouse model of *E. granulosus* infection by injecting either 2000 PSCs or an equal amount of PBS through the hepatic portal vein (n = 6). Mice were euthanized at 4-, 12-, and 32-week post-infection to examine the immune response and pathological changes at different time intervals. The establishment of a mouse model and the experimental design. We used the ELISA technique to quantify the levels of IL-9 in the blood serum of mice at different time intervals (4 weeks, 12 weeks, 32 weeks) (**A**) (n = 6). Figure (**B**) displays the results. We also performed RT-qPCR analysis to examine the expression levels of TLR2 (**C**), MyD88 (**D**), and NF-κB p65 (**E**) mRNA in the livers of mice at different stages of infection (n = 6). To assess pathological changes and protein expression, we performed H&E, PAS, and IHC staining (**F**) on mouse liver tissue sections. The liver tissue samples were analyzed to determine the expression levels of TLR2 (**G**), MyD88 (**H**), and NF-κB p65 (**I**) by IHC staining (n = 6). The percentage of positive regions was calculated to evaluate the expression levels. Statistics are displayed as the mean ± SD. * *p* < 0.05, ** *p* < 0.01 and **** *p* < 0.0001.

In this investigation, we obtained liver samples from female BALB/C mice infected with *E. granulosus* for durations of 4, 12, and 32 weeks. Additionally, we established uninfected control groups corresponding to each experimental group. The procedure for creating the mouse experimental model is described in Figure 2A. The findings of our study indicate a significant increase in serum IL-9 levels in infected mice over time compared to normal mice. This difference is visually represented in Figure 2B. The expression levels of TLR2 (Figure 2C), MyD88 (Figure 2D), and NF-κB p65 (Figure 2E) were evaluated comparatively. The study revealed that the levels of these proteins exhibited a more pronounced disparity as the duration of infection increased. Additionally, the diseased sections of mice were subjected to histological examination using H&E, PAS, and IHC staining. The findings are summarized in Figure 2F. The IHC results indicated predominant expression of TLR2 (Figure 2G), MyD88 (Figure 2H), and NF-κB p65 (Figure 2I) in the infected mice. As the infection progressed, there was a significant increase in the expression of these molecules. Compared to normal mice, expression levels began to rise at the 12th week of infection and showed a more pronounced increase by the 32nd week. These findings suggest that as the duration of infection increases, the activation of the TLR2/MyD88/NF-κB signaling pathway may play a role in various phases of infection. The observed increase in the activity of this signaling pathway may be associated with worsening liver damage, suggesting its involvement in disease progression.

### 3.3. Injection of Anti-IL-9 Reduced TLR2 in E. granulosus-Infected Mice

**Figure 3:**
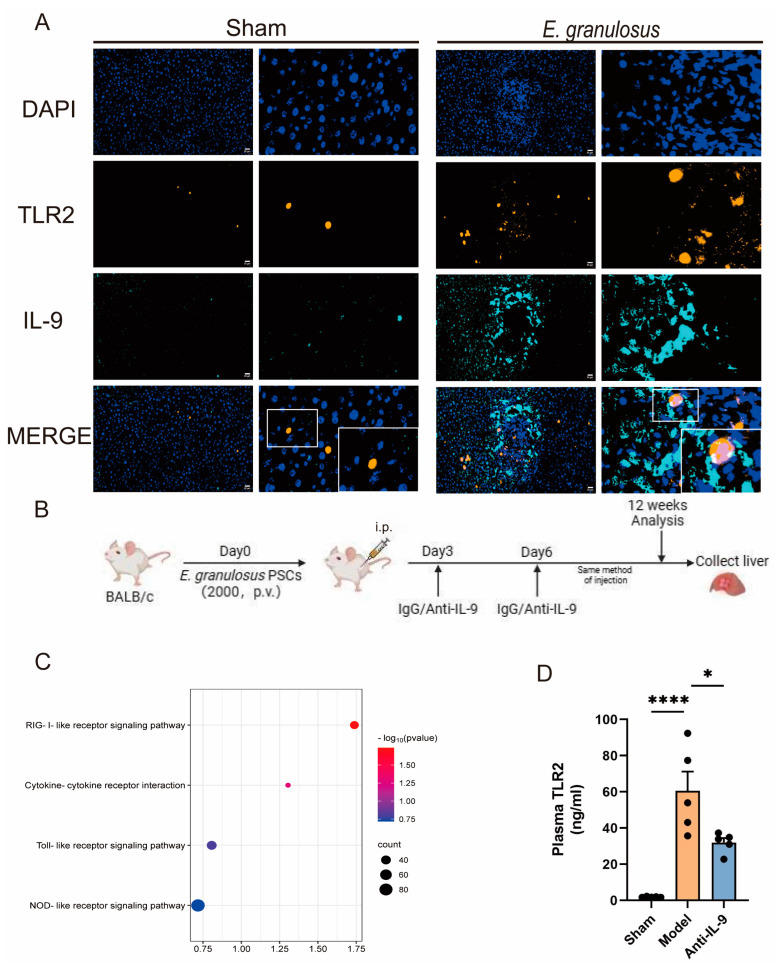
Injection of Anti-IL-9 reduced TLR2 in *E. granulosus*-infected mice. Our mIHC studies show that cells near of the lesion can be simultaneously labeled with DAPI (used for nuclear labeling), TLR2, and IL-9 on the same tissue slide (**A**). These results indicate a strong correlation between the expression of TLR2 and IL-9 and nuclear dispersion. In the experiment, female BALB/c mice were infected by injecting 2000 PSCs directly into the liver or an equal volume of PBS. After three days, the mice were randomly assigned to either the model or intervention group. The model group received intraperitoneal injections of the same volume of IgG, while the intervention group received injections of anti-IL-9. Injections were given at 3-day intervals for up to 12 weeks, followed by the euthanasia of the mice (**B**). Using the KEGG database for protein sequencing of the liver of mice injected with anti-IL-9, we discovered a remarkable association with the Toll-like receptor pathway (**C**). This suggests that IL-9 may modulate the immune response by affecting this specific pathway. We also quantified TLR2 levels in the plasma of three groups of mice using ELISA. The results showed a significant decrease in TLR2 levels in the plasma of mice injected with anti-IL-9 compared to the model group (*p* < 0.05). This suggests that Anti-IL-9 may reduce the immune response by decreasing the level of TLR2 (**D**) (n = 6). Statistics are displayed as the mean ± SD. * *p* < 0.05, **** *p* < 0.0001.

The objective of this study was to assess the potential impact of anti-IL-9 on the expression and production of the TLR2 signaling pathway. The investigation began with the treatment of *E. granulosus*-infected mice, which were subsequently subjected to a series of IHC analyses. Our observations revealed a co-localization of TLR2 and IL-9 in tissue sections, with notably increased expression near the lesions compared to control animals (Figure 3A). Subsequently, female BALB/c mice were infected with *E. granulosus*. The mice were then administered an intraperitoneal injection of anti-IL-9 antibodies, while a control group received IgG antibodies. This established an experimental model (Figure 3B). A protein sequencing analysis was conducted on the mouse liver. The KEGG pathway analysis revealed a strong correlation between the observed alterations in protein expression and the Toll-like receptor signaling pathway (Figure 3C). Furthermore, the concentration of TLR2 in the mice’s peripheral blood was measured (Figure 3D). A comparison of the control group with the anti-IL-9 treated mice revealed a notable reduction in the levels of TLR2 in the plasma of the latter (*p* < 0.05). This suggests that anti-IL-9 may exert a modulatory influence on the TLR2 signaling pathway (*p* < 0.05).

**Figure 4:**
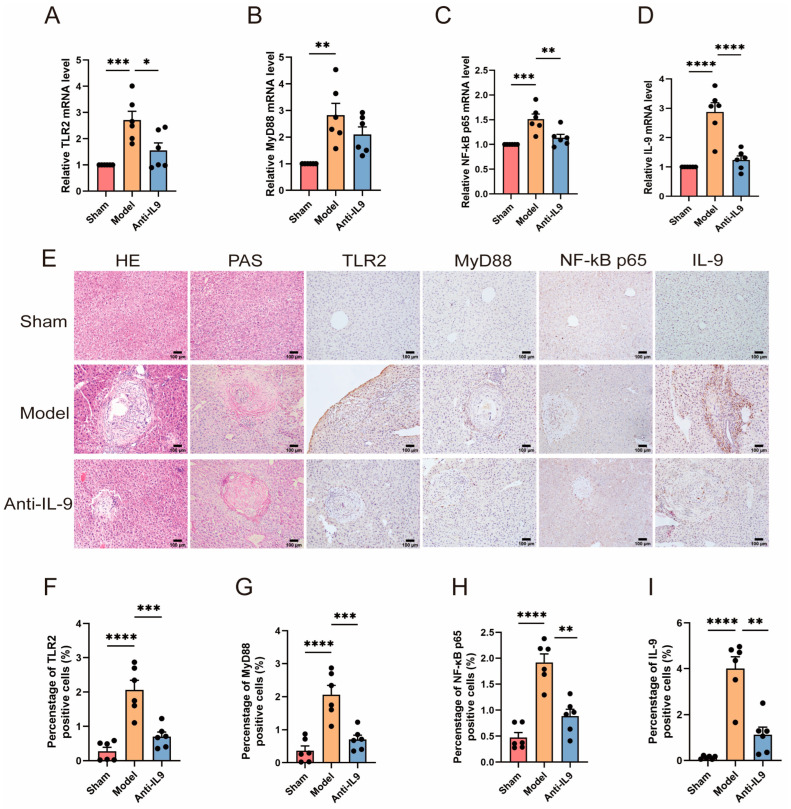
Injection of anti-IL-9 reduced TLR2 in *E. granulosus*-infected mice. To investigate this further, we used RT-qPCR to evaluate the relative expression levels of TLR2 (**A**), MyD88 (**B**), NF-κB p65 (**C**), and IL-9 (**D**) mRNA in several experimental groups (n = 6). Furthermore, IHC, H&E, and PAS staining techniques were used on mouse liver tissue sections to assess both pathological changes and protein expression levels (**E**). The hepatic expression levels of TLR2 (**F**), MyD88 (**G**), NF-κB p65 (**H**), and IL-9 (**I**) were quantitatively assessed by determining the proportion of positive cells in IHC staining (n = 6). Statistics are displayed as the mean ± SD. * *p* < 0.05, ** *p* < 0.01, *** *p* < 0.001 and **** *p* < 0.0001.

RNA was extracted from the livers of three groups of mice, and the relative mRNA expression levels of TLR2 (Figure 4A), MyD88 (Figure 4B), NF-κB p65 (Figure 4C), and IL-9 (Figure 4D) were quantified using qRT-PCR. The results demonstrated a significant decrease in the expression of IL-9 in the intervention group (mice treated with anti-IL-9) compared to the model group. This decrease was particularly pronounced in the IL-9 group (*p* < 0.001). Furthermore, histological examination was conducted on the diseased sections of mice using H&E, PAS, and IHC staining (Figure 4E). IHC staining demonstrated that animals treated with anti-IL-9 exhibited a significantly reduced positive area rate of the aforementioned immunological indicators compared to the model group (*p* < 0.01) (Figure 4F–I). H&E staining of the liver in mice from the model group revealed localized hepatocyte necrosis and extensive granuloma formation. However, the intervention group exhibited a reduction in hepatocyte damage and granuloma formation. Furthermore, PAS staining revealed that the intervention group exhibited mitigated hepatic glycogen loss compared to the model group, indicating a slower decrease in glycogen content. These findings suggest that the administration of anti-IL-9 may potentially result in a decrease in the expression of the TLR2/MyD88/NF-κB signaling pathway in the liver of mice. This reduction in pathway activity may contribute to the proliferation of parasites and worsening of liver damage.

### 3.4. The Effects of Immunity and Fibrosis Play an Important Role in RAW264.7 and HSCs

**Figure 5:**
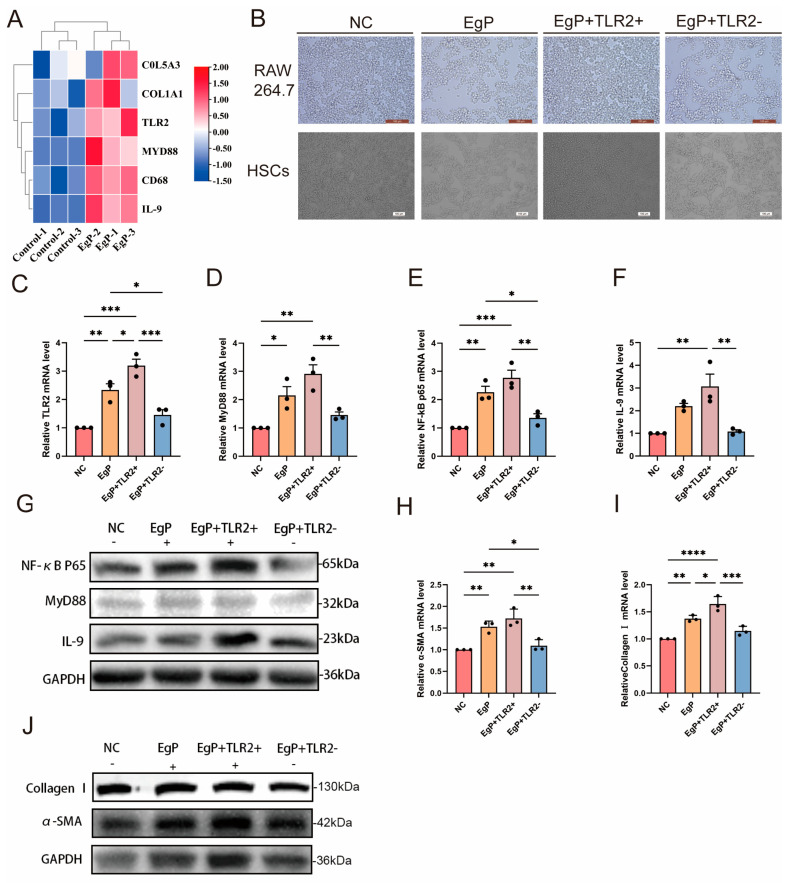
The effects of immunity and fibrosis play an important role in RAW264.7 and HSCs. We first incubated RAW264.7 macrophages and hepatic stellate cells (HSCs) with 15 µg/mL of EgP for 24 h. Next, 2 µL of Diprovocim and 2 µL of C29 were added to the culture system. This process took 1 h, after which the cells were collected for further study. We used KEGG analysis to examine the histological results of mouse spleen lymphocytes induced by worm proteins. The results showed a strong correlation among the Toll-like receptor pathway, IL-9, the fibrosis markers COL1A1 and COL5A3, and the macrophage surface marker CD68 (n = 3) (**A**). We then used an inverted microscope to capture images of cells from different treatment groups to examine changes in cell shape (**B**). The expression of TLR2 (**C**), MyD88 (**D**), NF-κB p65 (**E**), and IL-9 (**F**) in RAW264.7 was analyzed by qRT-PCR (n = 3). The levels of TLR2, MyD88, NF-κB p65, and IL-9 in RAW264.7 cells were measured by western blotting (n = 3) (**G**). The mRNA expression levels of α-SMA (**H**) and Collagen I (**I**) in HSCs were investigated by qRT-PCR. The protein expression of α-SMA and Collagen I in HSCs was simultaneously measured by Western blotting (n = 3) (**J**). Statistics are displayed as the mean ± SD. * *p* < 0.05, ** *p* < 0.01, *** *p* < 0.001 and **** *p* < 0.0001.

The RAW264.7 cell line is a commonly used model for studying immunomodulatory activity. It is frequently employed to imitate and analyze the immune cell response to various substances. To examine the immunological response of RAW264.7 cells, varying doses of EgP were used to stimulate them. This replicated the conditions of an *E. granulosus* infection in vitro. Following the co-culture of EgP with mouse spleen lymphocytes, there was a notable elevation in the expression levels of immunological markers CD68 and TLR2. Furthermore, the levels of fibrosis markers COL1A1 and COL5A3 were also increased (Figure 5A). Following the intervention, the cells were observed under an inverted microscope to assess changes in cell morphology (Figure 5B). The most effective concentration of EgP was identified to be 15 μg/ml. EgP was co-cultured with mouse macrophages and hepatic stellate cells at this concentration for 24 h. After 24 h, the TLR2 agonist diplodocid was added, followed by the TLR2 inhibitor C29. The cells were harvested after one hour of treatment. The mRNA levels of TLR2 (Figure 5C), MyD88 (Figure 5D), NF-κB p65 (Figure 5E), and IL-9 (Figure 5F) were quantified using quantitative reverse transcription polymerase chain reaction (qRT-PCR). The mRNA levels of α-SMA (Figure 5H) and Collagen I (Figure 5I) were also quantified. Additionally, the expression of α-SMA was evaluated through western blot analysis. The activation of the TLR2 signaling pathway was confirmed by validating RAW264.7 (Figure 5G) and HSCs (Figure 5J) using western blot techniques. These findings indicate that EgP can trigger an immunological response in mouse macrophages through TLR2. This immune response may be facilitated by the activation of the TLR2/MyD88/NF-κB signaling pathway, leading to inflammation in RAW264.7 cells. Concurrently, the activation of HSCs may also result in fibrosis, thereby exacerbating liver damage.

## 4. Discussion

The liver is a favored site for the spread of numerous tumors and the colonization of viruses and parasites due to its distinctive immunological environment [32,33]. This environment provides the pathogen with optimal conditions for its proliferation. Hepatic echinococcosis is a parasitic disease that invades the liver, causing inflammation and disruption of the liver tissue’s natural structure [1,34]. The disturbance creates an unfavorable immunological milieu that facilitates the gradual and persistent expansion of the parasite, potentially leading to liver fibrosis and necrosis in the host. *E. granulosus* effectively evades the host’s immunological response by exploiting the innate immune system, thereby ensuring its own survival. Currently, the management of hepatic echinococcosis is primarily surgical, as pharmacological therapy is hindered by issues such as drug resistance and adverse reactions. Identifying alternative treatment methods for hepatic echinococcosis is therefore of utmost importance. Recent research has demonstrated that infection with *E. granulosus* leads to a notable upregulation of TLR2 and IL-9 expression. Previous research has demonstrated that the levels of TLR2 and IL-9 mRNA expression in PBMCs of patients with CE are significantly higher compared to HC individuals [22,35]. PBMCs are an integral component of the immune system, comprising macrophages, dendritic cells, and other cell types. They play an essential role in defending the body against parasites such as *E. granulosus* and other foreign substances. Macrophages and dendritic cells within the local area can regulate the production and release of inflammatory substances [36]. Infection by *E. granulosus* activates immunological pathways in the liver. However, this process also damages the immune system, significantly disrupting the liver’s ability to defend itself and worsening liver damage. Furthermore, the liver lesions in mice infected with *E. granulosus* exhibited distinctive characteristics. By the 12th week of infection, a significant number of hepatocytes had been destroyed, resulting in the release of inflammatory cells from the liver and the formation of cystic lesions. By the 32nd week, the cystic lesion had transformed into a calcified state. Additionally, the liver exhibited varying degrees of damage, calcification, and necrosis. The immunological index persistently increases, indicating a progressive impairment of the immune function in mice and a worsening of liver injury. The use of H&E and IHC labeling techniques revealed a progressive infiltration of inflammatory cells near the infection site as the infection progressed. Cystic formations were observed, especially at the 12th and 24th weeks of infection [37]. The transition from fibrotic foci to cystic foci to calcified foci suggests that the parasite is secreting inflammatory substances to prevent its elimination by the host, thus facilitating immune evasion.

Our research indicates that IL-9 functions not only as a Th2 cytokine but also as a regulatory factor in the *E. granulosus* infection process. In this study, we observed a reduction in the expression of the TLR2 signaling pathway in mice infected with *E. granulosus* following an intraperitoneal injection of anti-IL-9. The activation of either the Th1 or Th2 cell is typically involved most clinical consequences of *E. granulosus* infection. In the advanced stages of infection, the disease is typically influenced by Th2 responses, indicating vulnerability. IL-9 is a cytokine associated with different Th2 responses due to its ability to stimulate allergic inflammation [38,39]. Preliminary research indicated that mice receiving the anti-IL-9 vaccination demonstrated resistance to *Leishmania* infection, effectively preventing leishmaniasis and delaying disease progression [40]. The observation that mice, which typically expel worms, become vulnerable following the administration of anti-IL-9 indicates that IL-9 plays a crucial role in nematode infection. Furthermore, the administration of anti-IL-9 to mice that typically expel worms demonstrated a significant impact, making these mice susceptible to nematode infection. This indicates that IL-9 plays a pivotal role in susceptibility to nematode infection [41]. In this research, we administered anti-IL-9 to mice infected with *E. granulosus* via intraperitoneal injection. Subsequently, a reduction in the number of inflammatory cells and a decrease in the size of liver lesions were observed. However, this subsequently results in an increased proliferation of parasites, thereby exacerbating liver damage.

Macrophages are a fundamental component of the innate immune system, serving as the liver’s initial barrier against infections. Macrophages stimulate the immune system by activating PRRs, such as TLR2, which detect PAMPs from parasites. This leads to an immune response [42]. Previous studies have shown that macrophages play a vital role in granuloma formation, inflammation progression, and liver fibrosis in parasite infections [43]. In the mouse model infected with the parasite, macrophages play a crucial role in supporting parasite survival by actively manipulating the immune system to evade host detection [44]. TLR2 plays a pivotal role in detecting helminth products by dendritic cells and macrophages and in establishing Th2 responses [45]. TLR2 recognition of parasite components is essential for the initiation of a specific polarized T helper response, enabling parasites to evade the toxic immune responses and persist within the host [46]. Macrophages play a role in establishing an immunological milieu in the liver of *E. granulosus*-infected mice. During the initial stages of infection, macrophages exhibit a pro-inflammatory phenotype, facilitating parasite elimination. During long-term infection, environmental factors cause macrophages to adopt an anti-inflammatory phenotype, which facilitates the continuation of the infection [37]. The study demonstrated that the expression product of EgP modulated the TLR2 signaling pathway after a 24-h co-culture with RAW 264.7. Moreover, TLR2 interference resulted in alterations in IL-9 expression, indicating a regulatory influence on IL-9. Thus, it can be proposed that manipulating the TLR2 signaling pathway may influence IL-9 regulation. In particular, the application of the TLR2 inhibitor C29 may help reduce immunological damage in mouse macrophages.

Infection with *E. granulosus* can cause irreversible liver damage, predominantly in the form of chronic fibrosis. This lesion significantly deteriorates liver structure and function. HSCs are the primary cells responsible for the promotion of fibrosis in the liver. Upon activation, these cells differentiate into myofibroblasts, acquiring the capacity for proliferation, migration, and contraction. These cells produce a significant amount of extracellular matrix, leading to scar tissue formation and disruption of the liver’s sinusoid structure. Hepatic fibrosis is a progressive condition that develops in response to an increase intrahepatic pressure [47]. Preliminary research suggests that substances released by *E. granulosus* cysts may contribute to cystic liver fibrosis. Fibrosis surrounds the parasite cyst, restricting its growth and causing changes in the structure of the liver [48]. Our research has shown that the presence of EgP in co-culture with HSCs increases collagen secretion, exacerbating fibrosis. By administering C29, we observed a significant reduction in the extent of fibrosis. This suggests that C29 may reduce the fibrotic response of HSCs by regulating the TLR2 signaling pathway. The TLR2/MyD88/NF-κB signaling pathway, mediated by TLR2, is crucial for macrophage activation and stellate cell promotion, contributing to liver fibrosis progression.

This study suggests a possible link between TLR2 and IL-9 in *E. granulosus* infection. IL-9 is likely to have a significantly impact the regulation of the TLR2/MyD88/NF-κB signaling pathway in a mouse model of liver injury infected with *E. granulosus*. Investigating the relationship between TLR2 and IL-9 may provide new insights and approaches for the diagnosis and treatment of CE. A comprehensive understanding of these molecular interactions is crucial for the development of precise treatment approaches aimed at reducing liver damage and improving patient outcomes.

## Figures and Tables

**Table 1:** Clinical characteristics of enrolled subjects.

Group	HC	CE	*p*
Case	20	20	
Sex (male/female)	9:11	7:13	
Age (years)	37.65 ± 10.38	38.55 ± 18.22	
BMI	26.75 ± 3.55	22.85 ± 4.65	
Location (right/left)	0	5:15	
AST (U/L)	20.48 ± 4.51	29.52 ± 10.31	***
ALT (U/L)	20.75 ± 10.88	29.74 ± 16.18	*

CE: cystic echinococcosis; HC: healthy controls (* *p* < 0.05, *** *p* < 0.001).

**Table 2:** Quantitative PCR Primers.

Primers Name	Primers Sequences (5′ to 3′)		Length (bp)
Mus-TLR2	Forward	ATGCTTCGTTGTTCCCTGTGTTG	23
	Reverse	AGTGGTTGTCGCCTGCTTCC	20
Mus-MyD88	Forward	GCAGAACCAGGAGTCCGAGAAG	22
	Reverse	GATGCCTCCCAGTTCCTTTGTTTG	24
Mus-NF-κB p65	Forward	ATGGGAAACCGTATGAGCCTGTG	23
	Reverse	AGTTGTAGCCTCGTGTCTTCTGTC	24
Mus-IL-9	Forward	GATGCGGCTGATTGTTT	17
	Reverse	CTCGTGCTCACTGTGGAGT	19
Mus-α-SMA	Forward	TTCGTGACTACTGCCGAGC	19
	Reverse	GTCAGGCAGTTCGTAGCTCT	20
Mus-Collagen I	Forward	CCCTGGTCCCTCTGGAAATG	20
	Reverse	GGACCTTTGCCCCCTTCTTT	20
Mus-GAPDH	Forward	GGTTGTCTCCTGCGACTTCA	20
	Reverse	TGGTCCAGGGTTTCTTACTCC	21
Homo-TLR2	Forward	CTCCCAGCAGGAACATCTGCTA	21
	Reverse	CCAGGAATGAAGTCCCGCTTA	21
Homo-MyD88	Forward	CGCCGCCTGTCTCTGTTCTTG	21
	Reverse	GGTCCGCTTGTGTCTCCAGTTG	22
Homo-NF-κB p65	Forward	GCAGTTTGATGATGAAGACC	20
	Reverse	CTGTCACTAGGCGAGT	16
Homo-IL-9	Forward	GATCCAGCTTCCAAGTGCCACTG	23
	Reverse	AAGCATGGTCTGGTGCAGTTGTC	23
Homo-GAPDH	Forward	CAGGAGGCATTGCTGATGAT	20
	Reverse	GAAGGCTGGGGCTCATTT	18

## Data Availability

The raw data supporting the conclusions of this article will be made available by the corresponding author on request.

## Supplementary Materials

The following supporting information can be downloaded at: https://www.mdpi.com/article/10.3390/biom14081007/s1, Figure S1: Subject analysis of AST, ALT, and their correlation with TLR2 and IL-9, along with imaging pictures. Figure S2: Mine the role of the TLR2 signaling pathway in human diseases from various databases.
