# Supplementary material for: Association of IL-9 Cytokines with Hepatic Injury in Echinococcus granulosus Infection
Source: Biomolecules. 2024 Aug 14;14(8):1007. doi: 10.3390/biom14081007 (PMC11352830; doi:10.3390/biom14081007)

Supporting information

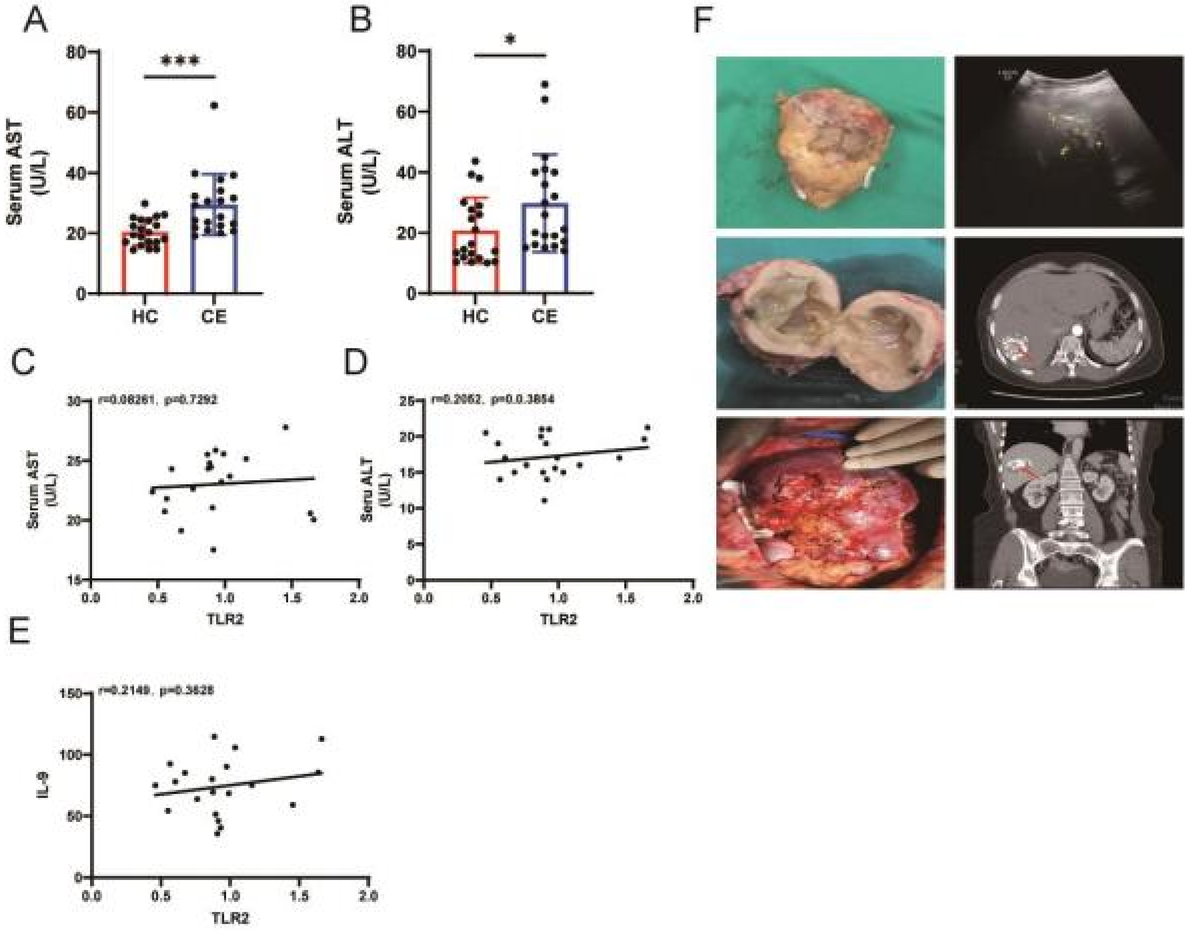

Figure S1. Serum levels of AST (A) and ALT (B) were analyzed individually in all patients (N = 20) to assess liver health. These two enzymes are often used as indicators of liver function. The aim of the study was to evaluate the association between blood levels of AST (C) and ALT (D) and TLR2 expression in 40 patients to investigate the possible involvement of TLR2 in liver disease. The correlation between TLR2 and IL-9(E). This investigation has provided insight into the functional relationship between TLR2 and immunological responses, as IL-9 is involved in immune control and inflammatory responses(E). We offer visual images of patients with CE and the associated diagnostic findings(F). These imaging data provide conclusive evidence for understanding the pathogenic characteristics and progression of CE.

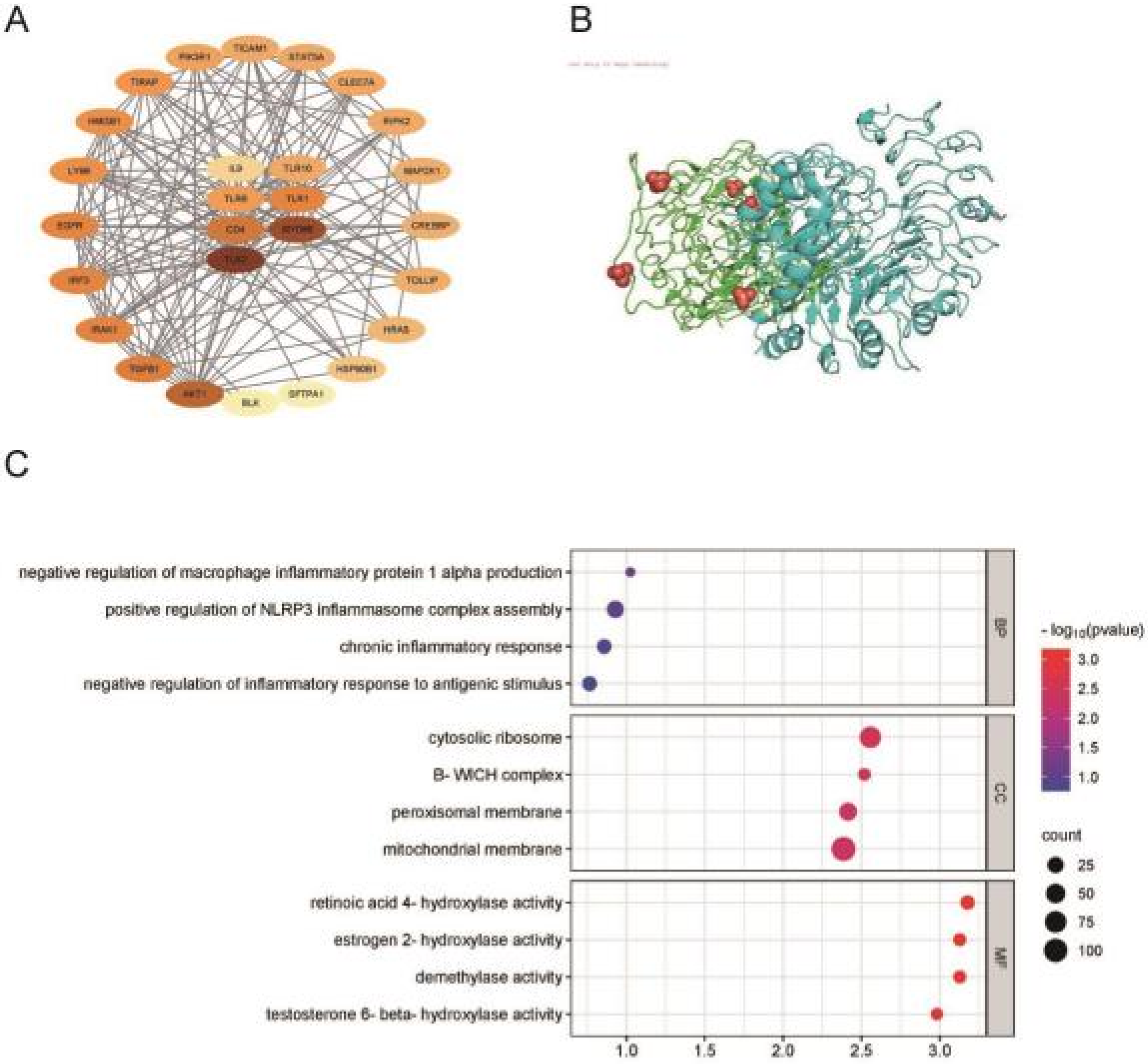

Figure S2. Protein-protein association network (PPI) analysis was used to investigate the association between TLR2, MyD88, and IL-9(A), providing insight into the collaborative function of these key immune molecules within cells. To further our understanding of the structural characteristics of these proteins, we carried out observations using three-dimensional protein structure maps (B). This three-dimensional perspective provides a clear understanding of the spatial organization and potential functional domains of proteins, which is crucial for revealing their involvement in the immune response. The KEGG database (C) was used to investigate the expression patterns of different genes. The KEGG analysis identified the involvement of these genes in biological pathways, providing a thorough understanding of their regulatory networks and signaling mechanisms in immune responses.

The following are the original images of Western blot.

RAW264.7

GAPDH

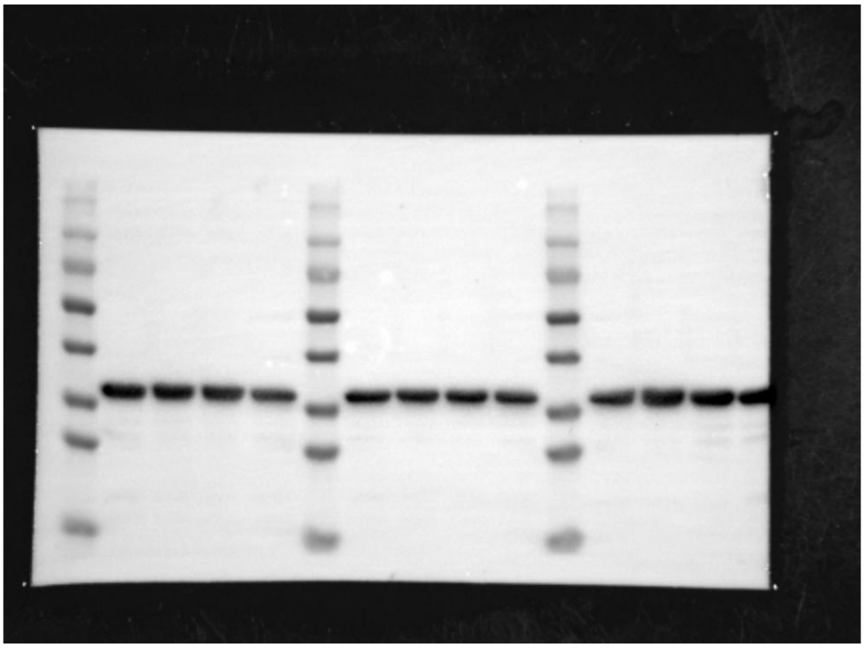

IL-9

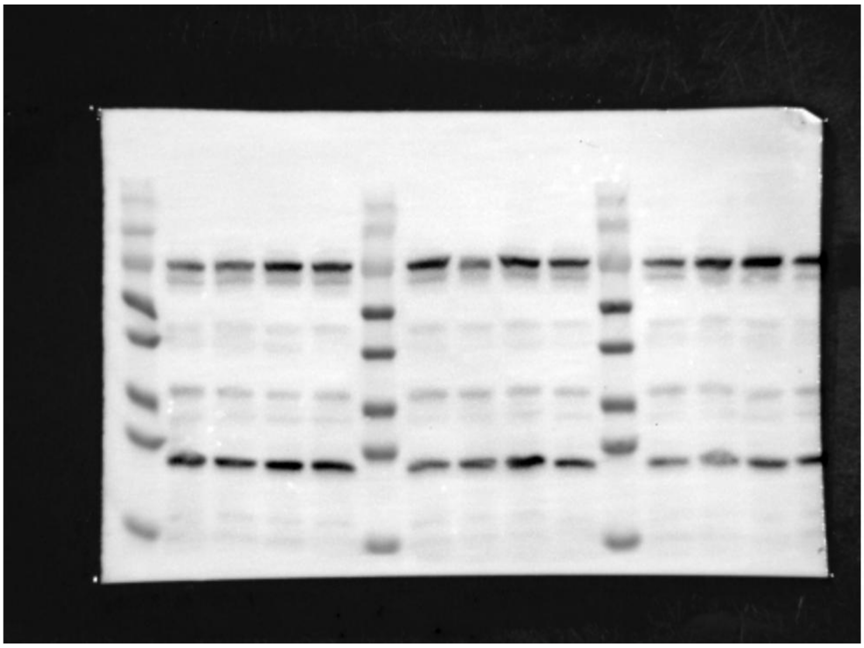

MyD88

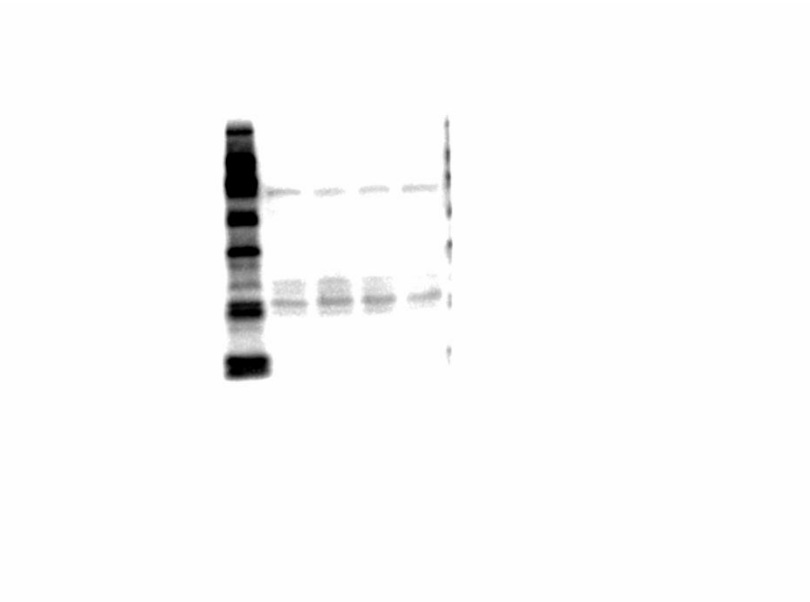

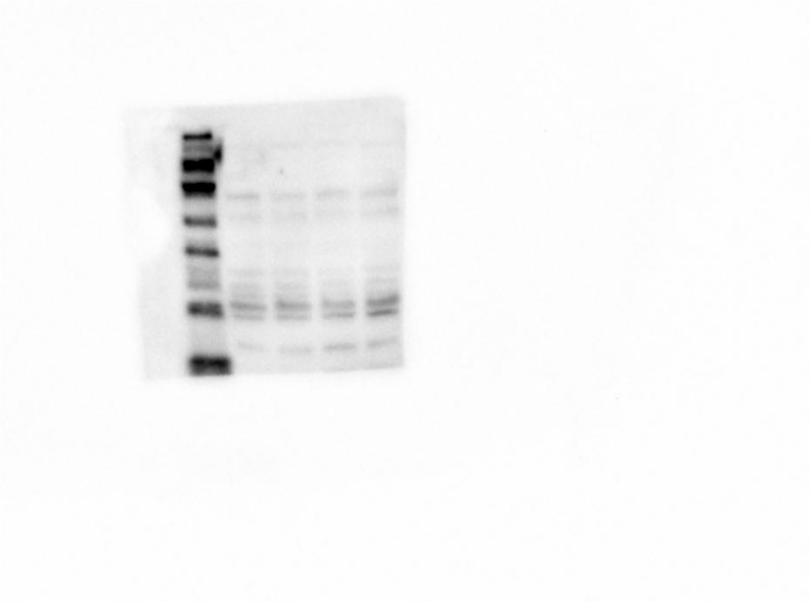

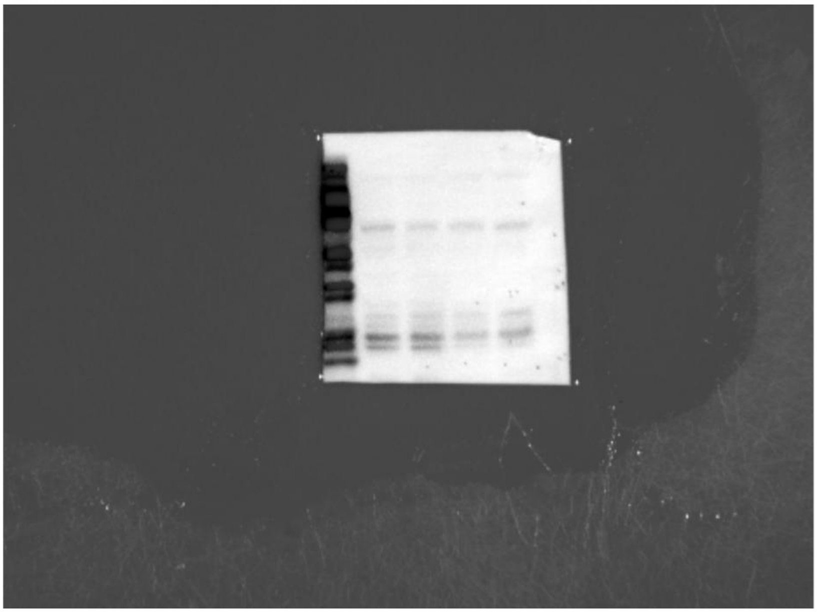

NF-kB p65

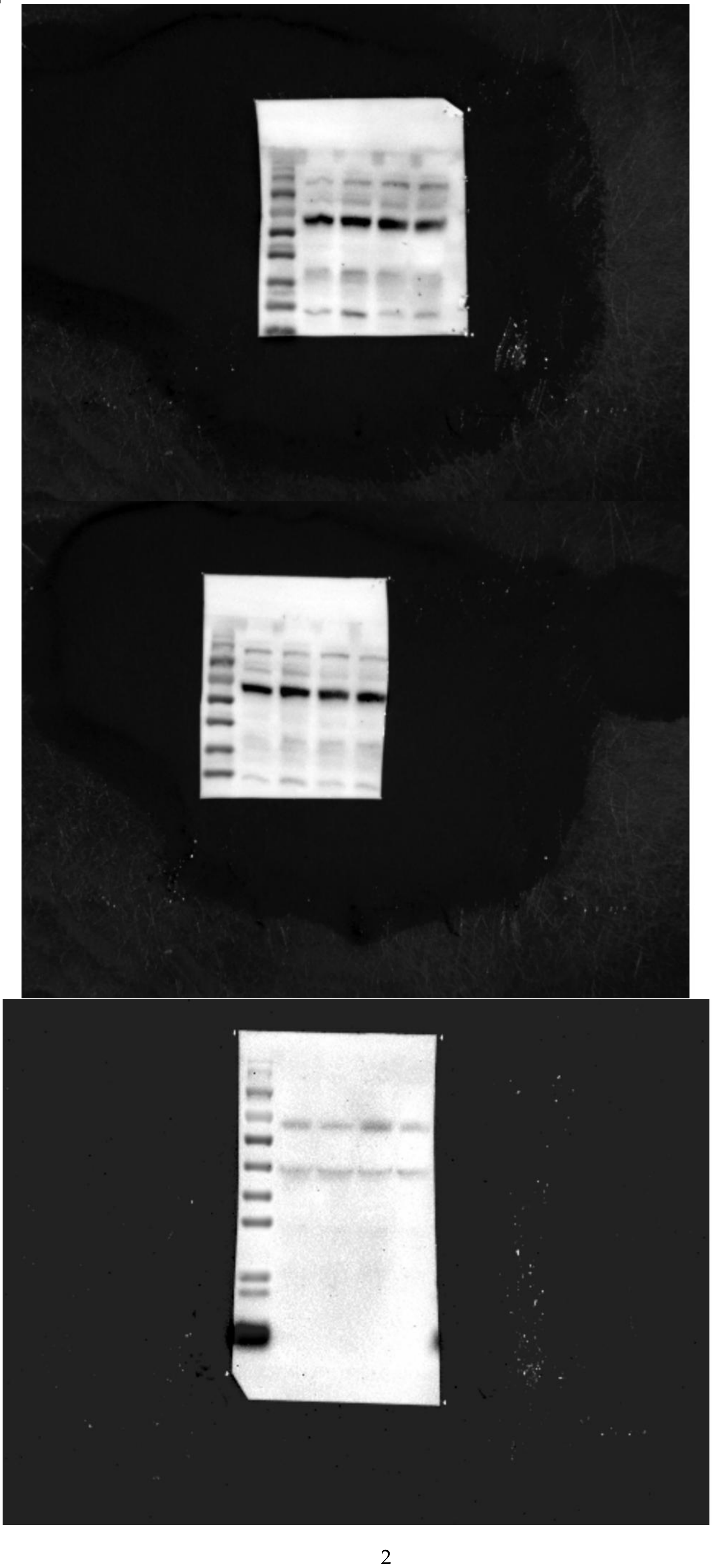

HSCs cells

a-SMA

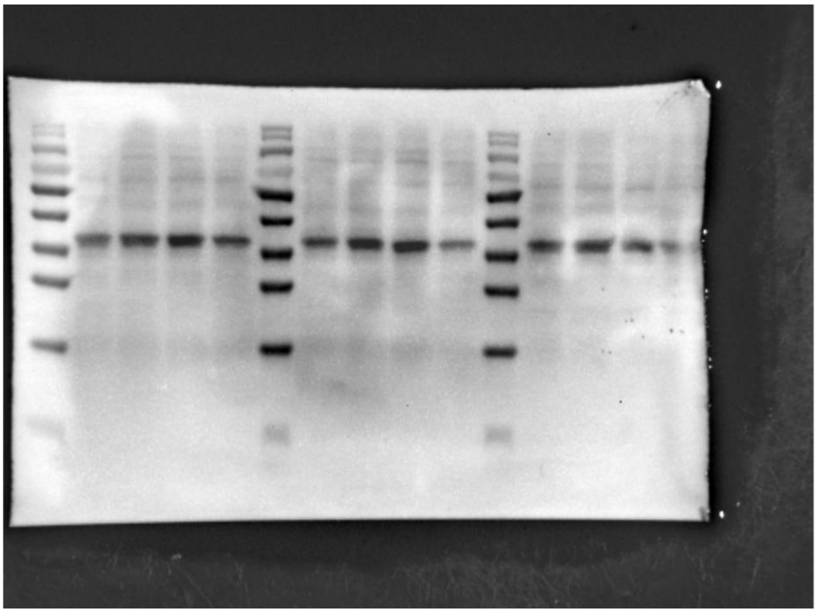

Collagen Ⅰ

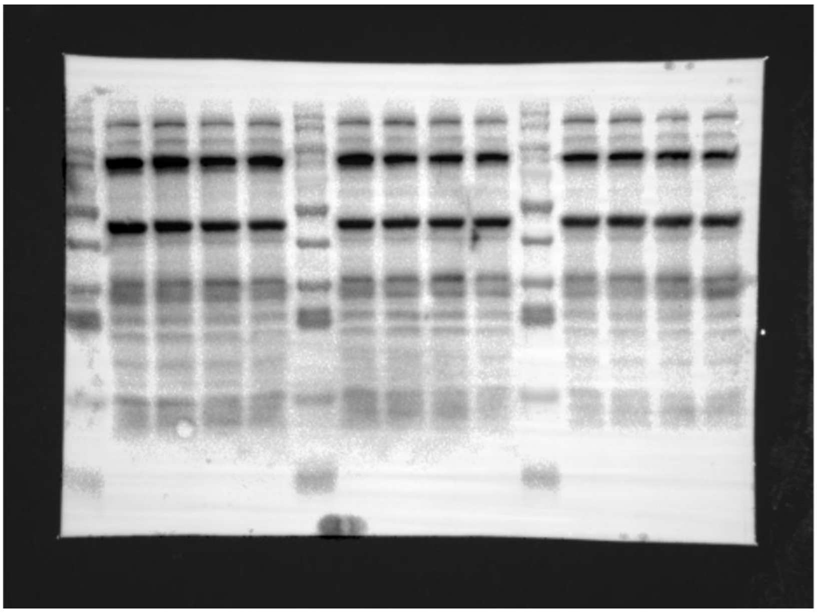

Supplement: Supplementary file 1 [file biomolecules-14-01007-s001.zip › biomolecules-3120022-supplementary.pdf]
